# Group Psychological Therapy for ADHD in Adults

**DOI:** 10.1192/j.eurpsy.2026.11728

**Published:** 2026-07-31

**Authors:** M. Lozano Madrid, L. Barbero, G. Vázquez, J. Guarch

**Affiliations:** Hospital Clínic, Barcelona, Spain

## Abstract

**Introduction:**

Attention Deficit Hyperactivity Disorder (ADHD) is a neurodevelopmental disorder with a prevalence of 2.5% in the adult population. Multimodal and combined interventions (pharmacological and psychological treatment) are the treatment of choice for ADHD in adults.

**Objectives:**

To develop a group psychological treatment protocol for adult patients diagnosed with ADHD. The aim is to reduce the core symptoms of the disorder and consequently improve overall functioning.

**Methods:**

This protocol is applied in a group format (between 12 and 16 participants) and follows a psychoeducational approach. It consists of 12 weekly sessions, each lasting 90 minutes. The treatment content is structured into four modules: organization and planning, distractibility, procrastination and impulsivity, and adaptive thinking. Two scales are administered before and after the intervention to assess its effectiveness in symptom reduction and its impact on disorder-related dysfunction: the Attention Deficit/Hyperactivity Disorder-Rating Scale (ADHD-RS) and the Weiss Functional Impairment Rating Scale-Self-Report (WFIRS-S). Inclusion criteria: patients over 18 years old with ADHD, evaluated by clinical psychologists or psychiatrists at Hospital Clínic de Barcelona. Exclusion criteria: unstable psychopathological comorbidity.

**Results:**

To date, the intervention has been conducted with two patient groups. A reduction in the interference of ADHD symptoms is expected as a result of acquiring compensatory adaptive strategies worked on during treatment.

**Conclusions:**

This protocol has been developed based on a review of the recommendations from Clinical Practice Guidelines and the adaptation of other evidence-supported non-pharmacological protocols and treatments. It is grounded in the principles of Cognitive Behavioral Therapy, which has shown strong evidence among psychological treatments for ADHD. Furthermore, the group format allows for an efficient and structured response to the needs of the population, while maintaining the depth and coherence of the therapeutic model. Additionally, it promotes the normalization of suffering, shared learning, and patient empowerment, thereby reducing unnecessary medicalization.

**Disclosure of Interest:**

None Declared

